# Long term healthcare costs of infants who survived neonatal necrotizing enterocolitis: a retrospective longitudinal study among infants enrolled in Texas Medicaid

**DOI:** 10.1186/1471-2431-13-127

**Published:** 2013-08-20

**Authors:** Vaidyanathan Ganapathy, Joel W Hay, Jae H Kim, Martin L Lee, David J Rechtman

**Affiliations:** 1 Department of Clinical Pharmacy and Pharmaceutical Economics & Policy, University of Southern California, 3335 S Figueroa Street, Unit A, Los Angeles, CA 90089-7273, USA; 2 Schaeffer Center for Health Policy and Economics, University of Southern California, Los Angeles, CA 90089-7273, USA; 3 Department of Pediatrics, University of California San Diego, San Diego, California, USA; 4 Prolacta Bioscience, City of Industry, CA 91746, USA

## Abstract

**Background:**

Infants who survive advanced necrotizing enterocolitis (NEC) at the time of birth are at increased risk of having poor long term physiological and neurodevelopmental growth. The economic implications of the long term morbidity in these children have not been studied to date. This paper compares the long term healthcare costs beyond the initial hospitalization period incurred by medical and surgical NEC survivors with that of matched controls without a diagnosis of NEC during birth hospitalization.

**Methods:**

The longitudinal healthcare utilization claim files of infants born between January 2002 and December 2003 and enrolled in the Texas Medicaid fee-for-service program were used for this research. Propensity scoring was used to match infants diagnosed with NEC during birth hospitalization to infants without a diagnosis of NEC on the basis of gender, race, prematurity, extremely low birth weight status and presence of any major birth defects. The Medicaid paid all-inclusive healthcare costs for the period from 6 months to 3 years of age among children in the medical NEC, surgical NEC and matched control groups were evaluated descriptively, and in a generalized linear regression framework in order to model the impact of NEC over time and by birth weight.

**Results:**

Two hundred fifty NEC survivors (73 with surgical NEC) and 2,909 matched controls were available for follow-up. Medical NEC infants incurred significantly higher healthcare costs than matched controls between 6–12 months of age (mean incremental cost = US$ 5,112 per infant). No significant difference in healthcare costs between medical NEC infants and matched controls was seen after 12 months. Surgical NEC survivors incurred healthcare costs that were consistently higher than that of matched controls through 36 months of age. The mean incremental healthcare costs of surgical NEC infants compared to matched controls between 6–12, 12–24 and 24–36 months of age were US$ 18,274, 14,067 (p < 0.01) and 8,501 (p = 0.06) per infant per six month period, respectively. These incremental costs were found to vary between sub-groups of infants born with birth weight < 1,000g versus ≥ 1,000g (p < 0.05).

**Conclusions:**

The all-inclusive healthcare costs of surgical NEC survivors continued to be substantially higher than that of matched controls through the early childhood development period. These results can have important treatment and policy implications. Further research in this topic is needed.

## Background

Neonatal necrotizing enterocolitis (NEC) is a gastrointestinal inflammatory condition in neonates that has a detrimental effect on the survival and long term development of affected infants. The disease is most commonly seen in premature infants, although up to 25% of NEC cases have been observed among full-term babies [1,2]. The overall incidence of necrotizing enterocolitis in the United States among infants born with birth weights < 1500 grams is estimated to be 7 – 12% [3-7]. The etiology of NEC is understood to be multi-factorial; ischemic injury and aberrant microbial colonization, among other factors, have been found to play a very important role in the disease process [1].

Initial management of NEC is often highly complicated and resource intensive. About 44-70% of neonates diagnosed with NEC show signs of advanced disease (commonly categorized as Bell’s stage III) that requires surgical management [3,8,9]. The economic burden of NEC has been estimated to be approximately 500 million to 1 billion US dollars annually [1,10]. These estimates are probably conservative given the strong evidence to support the many adverse consequences of surgical NEC beyond the initial hospitalization period. In particular, the outcomes of bowel resection among survivors of the procedure has been well studied and has been found to be associated with complications such as short gut syndrome and prolonged administration of parenteral nutrition [9,11,12]. There also is growing evidence for a possible link between surgical NEC and growth and neurodevelopmental impairment, leading to poor health outcomes in the long term [9,13-17]. The long term economic outcomes, such as healthcare or special education costs, of NEC survivors have not been well studied and there are very few published studies on this topic. There is a clear need for research on this topic to understand the full spectrum of disease burden, and also to evaluate the cost-effectiveness of novel therapeutic strategies that can mitigate the risk of developing NEC or reduce its impact on infant growth and development in the long term.

The primary goal of this study is to compare the healthcare costs between 6 and 36 months of chronological age, among survivors of medical and surgical NEC to that of matched infants without a diagnosis of NEC during birth hospitalization. This study was driven by the hypothesis that NEC survivors on average will continue to have greater demand for healthcare services throughout the early developmental period due to the increased risk of long term complications in these children as compared to similar infants without a diagnosis of NEC. Furthermore, the incremental demand for healthcare services will vary depending on the type of NEC (medical or surgical).

## Methods

This is a retrospective cohort study using claims database analyses. Infants enrolled in the Texas Medicaid fee-for-service program, born between January 2002 and December 2003, were selected for this study. Greater than 50% of live births in Texas were covered by Medicaid [18]. Also, unlike in other large states such as California, Illinois or New York, a significant proportion of Texas Medicaid clients were managed under traditional fee-for-service arrangements during the study period (2002 to 2006), making this data source ideal for our research [19].

Infants with NEC diagnoses were identified using a primary or secondary ICD-9-CM code of 777.50-53 in the hospital or physician claims at the time of initial hospitalization. NEC infants who had undergone procedures such as exploratory laparotomy involving bowel resection, peritoneal drainage, enterostomy with creation of stoma, etc. were defined as having “surgical NEC” (Table 1). NEC infants without claims for any of these surgical procedures were defined as having “medical NEC”. Based on the theoretical work of Rosenbaum & Rubin (1985) [20] and Imbens & Rubin [21] on the role of propensity score matching in the design of observational studies, each NEC infant was matched to infants without NEC diagnoses at a 1:10 ratio without replacement. Propensity scores were obtained from a stepwise logistic regression of NEC on infant demographic and birth characteristics at baseline including black race, gender, prematurity, born with extremely low birth weight (ELBW; defined as birth weight <1,000 g using the 5th digit of ICD-9 codes: 765.0x – 765.1x), and presence of any major birth defects, which was defined as having one or more of the following conditions: congenital heart disease (CHD) including patent ductus arteriosus (PDA), neural tube defects (NTD), hereditary CNS conditions and cleft lip or palate. The matching technique was implemented using the PSMatching algorithm in SAS 9.2. [22].

**Table 1:** Procedures and ICD-9-CM codes used to define infants with surgical NEC

**Surgical procedures**	**ICD-9-CM Codes**
Intestinal resection procedures	45.02-3, 45.1, 45.29, 45.3-4, 45.41, 45.49, 45.50-2, 45.60-3,
	45.70-9, 45.8, 46.99
Procedures related to stoma creation	46.0 – 46.64
Intestinal anastomosis	45.9 – 94, 46.73-79, 46.93-4
Exploratory laparotomy	45.0, 45.00, 54.11
Percutaneous abdominal drainage	54.91

The longitudinal inpatient, outpatient, physician and prescription claim files of all infants were followed from 6 months up to 3 years after birth. Follow-up beyond this period was restricted by the extremely small sample size (< 30 children) in the surgical NEC cohort. Descriptive tests for attrition were performed by comparing the sample characteristics of children retained in each group at the end of 36 months to the characteristics at baseline. The prevalence of chronic developmental health conditions in the NEC and control groups were studied over the follow-up period. The combined Mantel-Haenszel odds ratios [23] adjusted for ELBW status were reported for these conditions for the NEC and matched control groups. The hospital utilization, inpatient, ambulatory care including home healthcare, and the all-inclusive (grand total) healthcare costs in the NEC and matched control groups were evaluated descriptively. The total all-inclusive healthcare costs represent the inflation-adjusted amounts paid by Medicaid for inpatient, ambulatory care including home healthcare, prescription and professional services (all in 2009 US$).

Generalized linear regressions with a log link function and gamma distribution assumption were used to model the incremental costs for medical and surgical NEC over matched controls between 6–36 months of age [24]. Cluster robust standard errors were used to account for correlation within subject. Three different models were explored to estimate the total healthcare costs per six months: 1) allowing the impact of NEC to vary over time by fitting an interaction term between NEC type (no NEC, medical NEC and surgical NEC) and age-period (6–12, 12–24 and 24–36 months of age); 2) allowing both NEC and ELBW effects to vary over time; and 3) adding an interaction term between NEC type and ELBW status in addition to specifying the time-varying slope terms for NEC and ELBW status. Race, gender and hospitalization status in the previous period (Yes/No) were included as covariates in all 3 specifications. The three model specifications were tested for over-fitting using standard goodness-of-fit criteria such as the deviance and the AIC (Akaike information criteria). The average incremental costs of medical and surgical NEC over matched controls at each age-period were estimated using the margins command in STATA 11.0 (College Station, TX) and predicted costs were estimated [25]. Statistical significance of all descriptive between-group comparisons and model-based coefficients was determined using an alpha level of 0.05.

This study was approved by the Health Sciences Review Board at University of Southern California. 

## Results

**Figure 1:**
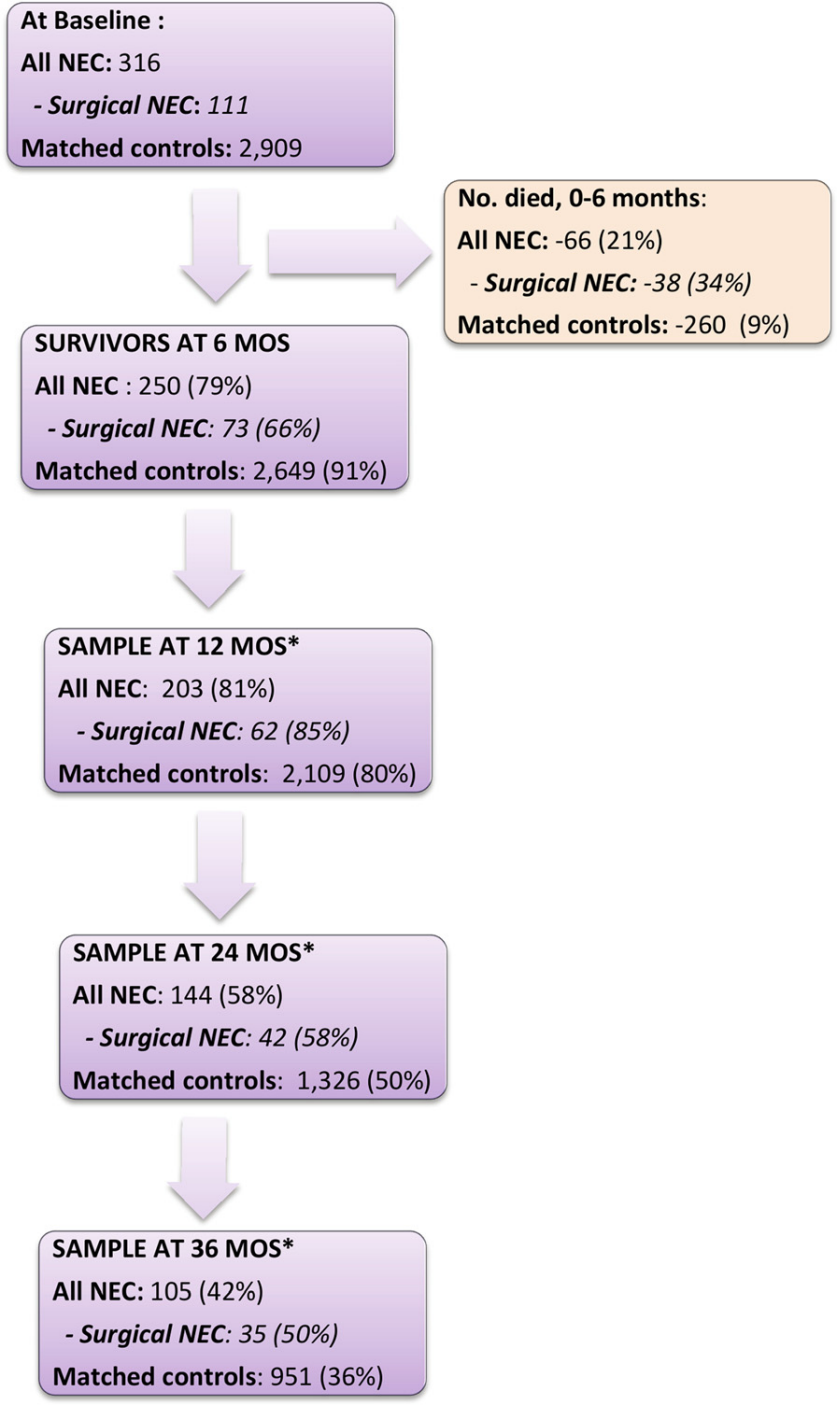
NEC and control group sample sizes (after matching) from 6 months to 3 years of age.

Three hundred and sixteen infants in the 2002–2003 fee-for-service Medicaid sample had NEC diagnoses, 111 of which had surgical NEC (Figure 1). Propensity matching at baseline resulted in 2,909 controls well-matched on all baseline characteristics except ELBW status, PDA and presence of neural tube defects (Table 2). There were 101 (32%) and 759 (26%) ELBW infants in the NEC and matched control groups, respectively (p < 0.05). PDA was the most common birth defect observed among NEC infants (30%) followed by neural tube defects (10%). The proportions of infants with PDA and neural tube defects among matched controls were 17% and 7%, respectively (p < 0.05). However, the two groups were comparable in the proportion of infants with any major birth defects as defined in the methods section (48% and 44% among NEC and matched controls, respectively, p = 0.13).

**Table 2:** Comparison of baseline characteristics between the NEC and control samples before and after propensity score matching

	**NEC**	**No NEC**	
**Characteristics**	**(n = 316)**	**Before matching**	**After matching**
		**(n = 122,929)**	**(n = 2,909)**
**Prematurity**	212 (67%)	15,451 (13%)**	1869 (64%)
**ELBW status**	101 (32%)	921 (0.8%)**	759 (26%)*
**Race**			
*African-American*	36 (11%)	7,777 (6%)**	350 (12%)
*White*	35 (11%)	13,964 (11%)	257 (9%)
*Hispanic*	111 (35%)	68,361 (56%)	1,407 (48%)
*Other / Unknown*	134 (43%)	32,827 (27%)	895 (31%)
**Male**	173 (55%)	62,252 (51%)	1,480 (51%)
**Birth defects**			
**Any major birth defects**^‡^	153 (48%)	8874 (7%)**	1,279 (44%)
*Patent ductus arteriosus*	95 (30%)	2284 (2%)**	501 (17%)**
*Neural tube defects*	33 (10%)	1101 (1%)**	200 (7%)*

*ELBW* extremely low birth-weight (BW <1000g), *NEC* necrotizing enterocolitis.

**p<0.01 and *p<0.05.

^**‡**^Includes congenital heart disease (CHD), neural tube defects, hereditary degenerative CNS conditions and cleft lip or palate.

Thirty-eight infants in the surgical NEC (34%), 28 in the medical NEC (14%) and 260 infants in the control groups (9%) died before 6 months of age (Figure 1). This left 250 NEC survivors (73 among them with surgical NEC) and 2,649 survivors among matched controls for follow-up beyond 6 months of age. Attrition was high among survivors in both NEC and control groups, mainly due to drop-out from the Medicaid program. Comparison of the characteristics of infants who were retained in the NEC and control groups at 36 months showed that there was no significant change within and between groups for the characteristics that were matched at baseline.

Table 3 lists the proportion of survivors in the medical NEC, surgical NEC and matched control groups with diagnoses of various chronic conditions observed during 6–12 and 24–36 months of age. Adjusting for ELBW status, the risk of developing bronchopulmonary dysplasia (BPD) was significantly higher in the medical and surgical NEC groups through 36 months of age (p < 0.01); the risk of malabsorption syndrome, metabolic disorders, failure to thrive (FTT) and neurodevelopmental delay (NDD) were significantly higher in the surgical NEC group than matched controls through 36 months of age (p < 0.05). Also, a significant difference was observed in the proportion of children receiving care for feeding difficulties and gastrointestinal ostomies between the surgical NEC and matched control groups through 36 months (p < 0.05). Medical NEC infants faced a significantly higher risk of FTT, feeding difficulties, NDD and open gastrointestinal ostomies between 6–12 months of age, but not in the subsequent periods of evaluation.

**Table 3:** Prevalence of chronic developmental health conditions in the NEC and matched control groups between 6–12 and 24–36 months of follow-up

***Age: 6–12 Months***					
**Chronic Conditions**	**Controls**	**Medical NEC**	**Surgical NEC**	**Adjusted odds ratio‡**	**Adjusted odds ratio‡**
	**(N = 2,109)**	**(N = 141)**	**(N = 62)**	**(95% CI)**	**(95% CI)**
	**n (%)**	**n (%)**	**n (%)**	**Medical NEC vs. Controls**	**Surgical NEC vs. Controls**
**BPD**	98 (5%)	19 (14%)	16 (26%)	3 (1.8 – 5.6)**	4 (2–7)**
**Malabsorption syndrome†**	13 (1%)	2 (1%)	13 (21%)	2.3 (0.5 – 10)	47 (19–116)**
**Failure to thrive**	132 (6%)	22 (16%)	18 (29%)	3 (1.7 – 5)**	4 (2–6)**
**NDD**	168 (8%)	21 (15%)	16 (26%)	1.9 (1.2 – 3.2)**	2.4 (1.4 – 4.5)**
**Cerebral palsy**	17 (1%)	3 (2%)	2 (3%)	2.6 (0.7 – 9)	2.3 (0.5 – 10.8)
**GI artificial openings present**	42 (2%)	9 (6%)	15 (24%)	3.2 (1.5 – 6.9)**	9 (5–21)**
**Metabolic disturbances**^**§**^	58 (3%)	5 (4%)	8 (13%)	1.2 (0.5 – 3)	4.8 (2.1 – 10.7)**
**Feeding difficulties**	33 (2%)	8 (6%)	14 (23%)	4 (1.7 – 8.4)**	11 (5.2 – 21.8)**
***Age: 24–36 Months***					
**Chronic Conditions**	**Controls**	**Medical NEC**	**Surgical NEC**	**Adjusted odds ratio‡**	**Adjusted odds ratio‡**
	**(N = 951)**	**(N= 70)**	**(N = 35)**	**(95% CI)**	**(95% CI)**
	**n (%)**	**n (%)**	**n (%)**	**Medical NEC vs. Controls**	**Surgical NEC vs. Controls**
**BPD**	14 (1%)	4 (6%)	5 (14%)	4.6 (1.4 – 15)**	5.5 (2–16)**
**Malabsorption syndrome†**	4 (0.4 %)	0 (0%)	7 (20%)	-	62 (15–249)**
**Failure to thrive**	97 (10%)	6 (9%)	11 (31%)	0.8 (0.3 – 2)	3 (1.3 - 6)*
**NDD**	133 (14%)	13 (19%)	13 (37%)	1.5 (0.8 – 2.9)	2.6 (1.2 – 5.6)*
**Cerebral palsy**	35 (4%)	1 (1%)	4 (11%)	0.4 (0.05 – 3)	2.1 (0.7 – 6.4)
**GI artificial openings present**	35 (4%)	4 (6%)	10 (29%)	1.8 (0.6 – 5.3)	6 (3–14)**
**Metabolic disturbances**^**§**^	29 (3%)	3 (4%)	4 (11%)	1.4 (0.4 – 5)	3.4 (1.1 -10)*
**Feeding difficulties**	19 (2%)	3 (4%)	4 (11%)	2.4 (0.7 – 9)	4 (1.2 - 13)*

*BPD* Bronchopulmonary dysplasia, *ELBW* extremely low birth weight, *GI* gastrointestinal, *NDD* Neurodevelopmental delay.

**p<0.01 and *p<0.05.

^**‡**^Odds ratios were obtained after adjusting for extremely low birth weight status using Mantel-Haenszel chi-squared tests.

^**†**^Malabsorption syndrome includes post-surgical non-absorption (ICD-9 579.3 commonly used to code for short bowel syndrome) and other unspecified intestinal malabsorption (ICD-9 579.9).

^**§**^Metabolic disturbances include disorders of amino acid, carbohydrate, lipid and mineral metabolism, etc. (ICD-9 codes 270–279).

The univariate distributions of healthcare utilization and costs in the NEC and matched control groups are presented in Table 4. Healthcare utilization and cost estimates were highly skewed and the bulk of utilization and healthcare costs were concentrated in the upper right tails in all 3 groups. Medical NEC infants on average had 3 additional hospital days than matched controls between 6–12 months of age (p < 0.01) but the corresponding inpatient costs did not reach significance between the two groups (p = 0.056). The total ambulatory care cost, including home healthcare costs, was significantly higher in the medical NEC group compared to matched controls for the 6–12 months of age period (p < 0.01). The difference in healthcare utilization and costs between medical NEC and control groups after 12 months of age was not statistically significant.

**Table 4:** Comparison of healthcare utilization and cost estimates (unadjusted) for medical, surgical NEC and matched control groups from 6 months to 3 years of age

**Age ->**	**6-12 months**			**12-24 months**			**24-36 months**		
	**Controls**	**Medical NEC**	**Surgical NEC**	**Controls**	**Medical NEC**	**Surgical NEC**	**Controls**	**Medical NEC**	**Surgical NEC**
N ->	2109	141	62	1326	102	42	951	70	35
**Mean Hospital**	0.1 (0.4)	0.2 (1)**	1 (1)**	0 (1)	0 (0.5)	1 (2)**	0 (1)	0 (0.3)	1 (1)**
**admissions **^**a **^**(SD)**									
IQR	0	0	1	0	0	1	0	0	1
90th percentile	0	1	2	1	1	3	0	0	1
**Mean Hospital days **^**b **^**(SD)**	1 (9)	4 (17)**	16 (33)**	1 (4)	1 (4)	10 (33)**	0.5 (3)	0.5 (3)	2 (6)**
IQR	0	0	10	0	0	4	0	0	2
90th percentile	2	6	59	2	3	13	0	0	3
**Inpatient costs (SD) **^**b**^	2,922 (28,056)	8,068 (33,452)	35,867 (79,511)**	1,942 (12,319)	2,557 (9,823)	23,102 (71,101)**	1,046 (8,737)	1,039 (6,395)	7,842 (29,930)**
IQR	0	0	24,150	0	0	7,589	0	0	2,615
90th percentile	2,167	8,769	126,901	3,435	6,296	26,085	0	0	11,786
**Total home healthcare**	741 (5,575)	2,882 (14,251)**	3,564 (9,895)**	2,539 (17,372)	3,102 (16,412)	11,364 (34,901)**	2,237 (14,806)	2,380 (13,167)	9,485 (23,184)**
^**b **^**costs (SD)**									
IQR	0	0	1,741	0	0	3,533	0	0	4,947
90th percentile	160	1,536	7,761	184	386	36,309	199	128	31,976
**Total ambulatory care**	2,332 (7,388)	5,129 (16,391)**	8,764 (14,920)**	5,961 (21,091)	6,392 (18,719)	21,715 (38,769)**	4,346 (19,294)	3,946 (14,009)	16,461 (34,291)**
**costs**^**b,c **^**(SD)**									
IQR	1,247	3,414	10,572	2,154	4,144	21,573	1,372	1,874	11,320
90th percentile	5,958	10,846	20,126	14,984	17,382	61,962	5,691	4,357	61,559
**Overall healthcare**	5,598(30,654)	13,610 (38,264)**	45,213 (87,497)**	8,726 (28,039)	9,856 (23,111)	46,378 (91,535)**	6,279 (24,018)	5,809 (16,966)	26,055 (52,637)**
IQR	2,238	7,157	41,957	3,987	8,921	32,077	2,466	1,706	17,753
90th percentile	8,661	25,432	143,132	18,075	22,827	106,997	8,779	10,290	84,338

*SD* standard deviation, *IQR* interquartile range (difference between the 75th and 25th percentile estimates).

*p<0.05 and **p<0.01.

a) Outcome was assumed to be negative binomial distributed while evaluating statistical significance of difference estimates between NEC versus control groups. b) Outcome was assumed to be t-distributed while evaluating statistical significance of difference estimates between NEC versus control groups. c) Total ambulatory care costs included costs of laboratory, medical consultation, preventive care, ambulatory surgery, home health services and professional payments. Overall healthcare costs include inpatient, ambulatory as well as prescription medication costs.

Surgical NEC survivors had significantly higher inpatient utilization and inpatient costs than matched controls during all time periods (Table 4). The difference in the mean unadjusted inpatient costs between surgical NEC and control groups was US$ 32,945, 21,160 and 6,796 per child corresponding to the difference in mean hospital days of 15, 9 and 1.5 days for the periods 6–12, 12–24 and 24–36 months of age, respectively (p < 0.01). The mean unadjusted ambulatory care cost among surgical NEC survivors was US$ 6,432, 15,754 and 12,115 more than that for matched controls, between 6–12, 12–24 and 24–36 months of age, respectively (p < 0.01). Home healthcare costs accounted for 44 to 60% of the difference in ambulatory care costs observed over the follow-up period between surgical NEC and control groups. The difference in the mean unadjusted all-inclusive healthcare costs between surgical NEC and control groups was US$ 39,615, 37,652 and 19,776 for the period 6–12, 12–24 and 24–36 months of age, respectively (p < 0.01).

Multivariate modeling demonstrated that the incremental effect of the two NEC types and ELBW status on the all-inclusive healthcare cost decreased over time (p < 0.01 for the slope terms NEC type and ELBW status by age-period). The healthcare cost models also predicted a decrease in the incremental effect of NEC on healthcare costs in ELBW infants (p = 0.09 and 0.02 for the interaction effect of medical and surgical NEC, respectively, with ELBW status). The specification that included all three interaction terms in the model (as shown below) had a superior fit over the other models tested, and, therefore, was used to obtain the adjusted incremental healthcare cost estimates of each NEC type over matched controls.

$$\text{Healthcare costs}\;/6\;\text{months}\;\alpha \;f\;(\begin{array}{l} \text{NEC type, ELBW status, race, gender, age-period, NEC type}\;X\\ \;\text{age-period, ELBW status}\;X\;\text{age-period, NEC type}\;X\;\text{ELBW status, prior hospitalization} \end{array}\;)$$

**Figure 2:**
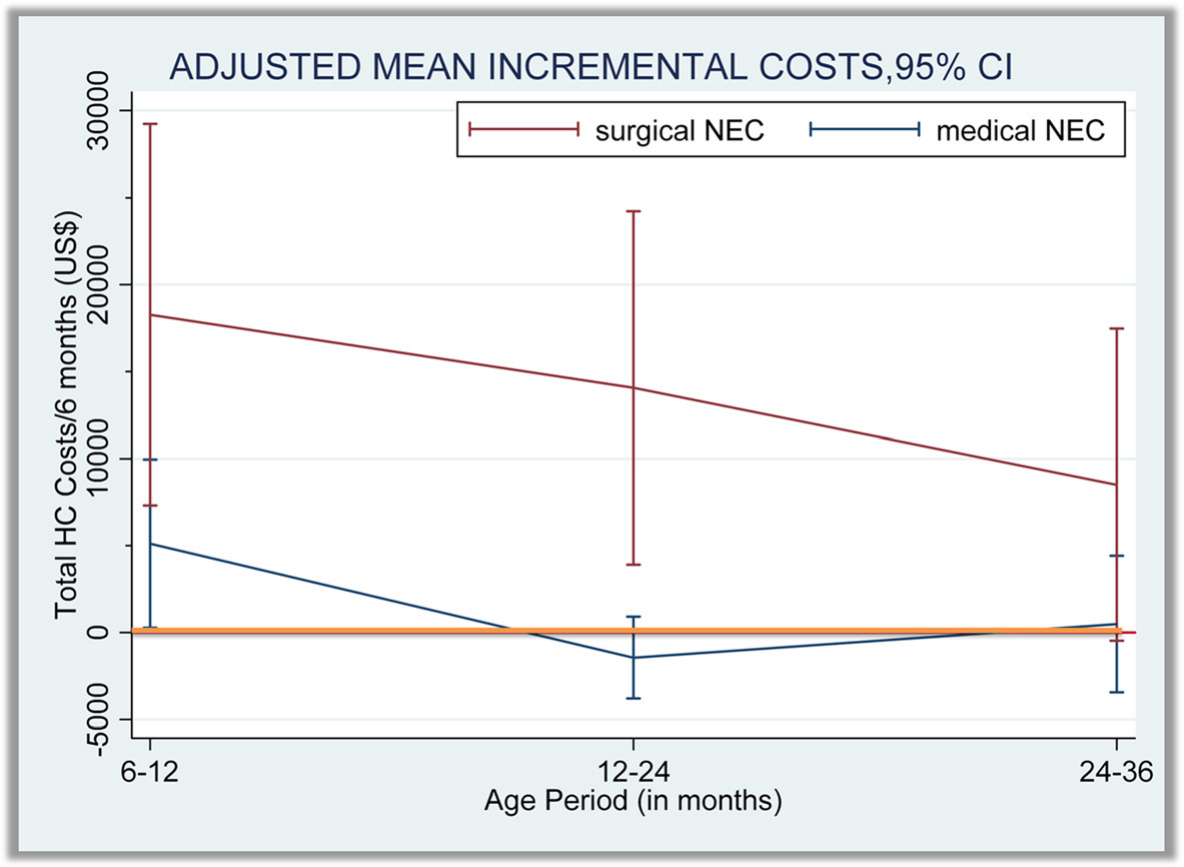
Adjusted incremental total healthcare costs per 6-months incurred by medical and surgical NEC survivors over matched controls from 6 months to 3 years of age.

**Figure 3:**
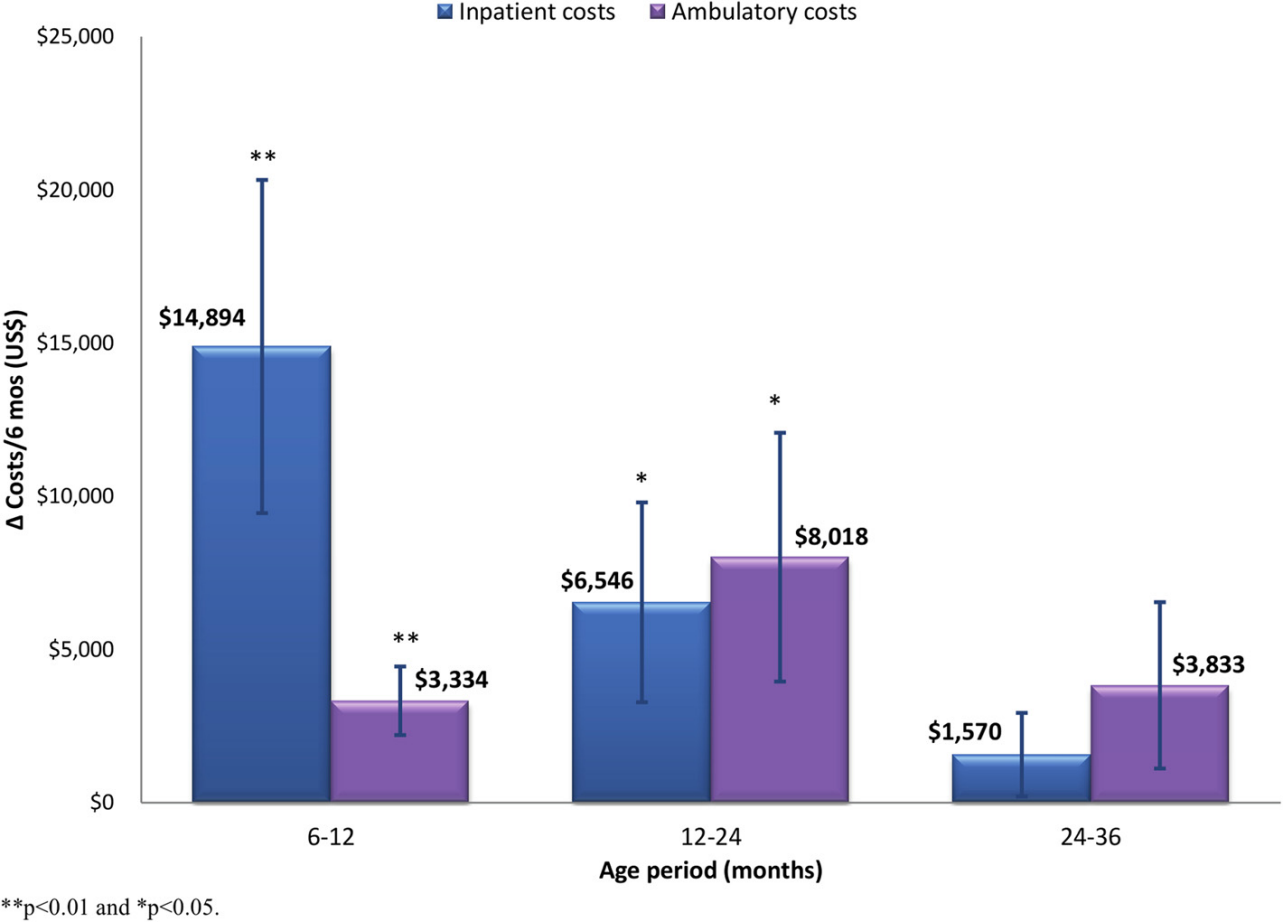
Adjusted incremental inpatient and ambulatory care costs per 6-months (± SE) incurred by surgical NEC children over matched controls, 6 months to 3 years of age.

The model-adjusted difference in all-inclusive healthcare cost between each NEC type and matched controls is shown in Figure 2. The adjusted mean incremental cost of medical NEC survivors was $5,112 between 6–12 months of age (95% confidence interval (CI): $274 - $9,950; p < 0.05). The adjusted healthcare cost difference between medical NEC and matched control groups was not statistically significant after 12 months of age. The adjusted mean incremental healthcare cost per six months in the surgical NEC group over matched controls was US$ 18,274 (95% CI: 7,315 - 29,234; p < 0.01), 14,067 (95% CI: 3,906 - 24,228; p < 0.01) and 8,501 (95% CI: -475 - 17,448; p = 0.06) for the periods 6–12, 12–24 and 24–36 months of age, respectively. Ambulatory care cost was the main driver of the healthcare cost differences between surgical NEC and control groups beyond 12 months of age as shown in Figure 3. The predicted mean healthcare costs of surgical NEC survivors and matched controls among sub-groups of children born with birth-weights <1,000g and ≥ 1,000g are reported in Table 5.

**Table 5:** Predicted costs per 6-months for surgicalNEC children versus matched controls over time across birth-weights

	**Birth-weight <1000g**			**Birth-weight ≥ 1000g**		
**Age**	**6-12**	**12-24**	**24-36**	**6-12**	**12-24**	**24-36**
**Control gp.**						
Predicted costs	8,540	15,559	10,347	1,526	3,795	3,356
95% CI (low)	6,210	11,830	7,287	1,086	3,026	2,156
95% CI (high)	$10,869	19,289	13,407	1,966	4,565	4,554
**Surgical NEC gp.**						
Predicted costs	34,020	27,013	14,476	17,311	18,764	13,367
95% CI (low)	12,597	9,329	2,945	5,603	7,158	3,143
95% CI (high)	55,443	44,696	26,006	29,020	30,371	23,590

## Discussion

Neonatal necrotizing enterocolitis has a high fatality rate among infants affected by the condition. The morbidity and long term health outcomes among NEC survivors are highly influenced by the pathological stage of NEC and the extent of damage to the intestines [11,26-28]. Surgical intervention is an important surrogate for severity of NEC and the associated high risk of mortality and poor developmental outcomes, regardless of the surgical procedure used [9-16,29-32]. Previous studies of healthcare costs associated with NEC have shown that both medically and surgically treated NEC infants incur significantly higher inpatient hospital expenditures than similar infants without NEC due to longer length of stay in neonatal intensive care units [10,33-35]. However, the healthcare costs of NEC survivors over the long term have not been studied to date despite increasing evidence of poor health outcomes among these children.

In this study we compared the healthcare utilization of 250 NEC survivors in the Medicaid population to that of controls matched on prematurity and ELBW status, black race and presence of birth defects, from 6 months to 3 years of age. When matching NEC infants to controls, we found that patent ductus arteriosus was more frequently observed in the NEC group. The association of PDA and NEC is well known and is thought to be due to excessive left to right shunting leading to systemic hypoperfusion, a known risk factor for NEC [36]. We found that 28 infants in the NEC group (9%) and 119 infants in the matched control group (4%) had undergone surgical PDA ligation or division procedures. While additional costs due to PDA surgery can be incurred during the initial hospitalization period, the cost of PDA over the long-term should be no different in infants with or without NEC. In other words, a PDA-NEC association that was observed in the data would not have an impact on the main findings of this paper.

We found that medical NEC survivors incurred $5000 more in healthcare costs on average than matched controls between 6 to 12 months of age. These incremental costs were mainly driven by ambulatory care expenses, possibly attributable to management of artificial GI openings and follow-up care received for other developmental problems observed during this period. These included failure to thrive, feeding difficulties, BPD and NDD. However, the healthcare costs of medical NEC survivors did not differ from matched controls after 12 months of age. These results indicate that the likelihood of experiencing developmental complications leading to increased utilization of healthcare resources over the long term (> 1 year of age) is not significant in comparing medical NEC survivors to matched controls.

On the other hand, the all-inclusive healthcare costs among surgical NEC survivors continued to be higher than matched controls beyond 6 months with the adjusted incremental costs being statistically significant up to 2 years of age. The incremental costs of surgical NEC between 2 to 3 years were still substantial and the lack of statistical significance could be due to the very small number of surgical NEC infants remaining in this time period (n = 35). Our findings show that surgical NEC survivors incurred an average $60,000 more in healthcare costs than matched controls over the period from 6 months to 3 years of age.

The high costs among surgical NEC survivors were initially driven by inpatient expenditures. However, the frequency of hospital admissions and level of inpatient expenditures decreased over time. Home healthcare and other ambulatory care expenditures were the main drivers of costs among surgical NEC children from 1 to 3 years of age. The net difference in costs between surgical NEC children and matched controls was smaller in the extremely low birth weight group (BW <1,000g) compared to the cost difference found in children born with BW ≥ 1,000g. A similar trend was noted in the risk of chronic health conditions wherein the odds ratios for the association of surgical NEC with developmental health conditions were smaller for infants with BW < 1,000g compared to BW ≥ 1,000g (though only the combined odds ratios were reported due to the very small number of NEC infants with BW < 1,000g). These results are to be expected given the already higher rate of complications in extremely low birth weight status and the consequent decrease in the marginal effect of surgical NEC on healthcare costs in these children. Nevertheless, the results clearly show that children born with extremely low birth weight and who survive severe NEC incurred higher healthcare costs than children with only one of these risk factors.

The intensity of healthcare use and costs among surgical NEC survivors could be driven by one or more factors such as: treatment for post-surgical complications (e.g., short bowel syndrome (SBS) / intestinal malabsorption which was seen more often in the surgical NEC group), costs associated with nutrition (e.g., length of total parenteral nutrition (TPN) support required by survivors and the complications associated with TPN), treatment of infections associated with ostomies (a significantly higher proportion of surgical NEC survivors lived with open ostomies for a significant period of time), and costs of care for very frequently reported conditions such as failure to thrive, NDD and nutritional and metabolic disturbances.

In a study among infants with short bowel syndrome, Spencer, et al. reported the average costs of SBS between year 1 to year 5 to be US$ 250,000-300,000 per year and that parenteral nutrition alone contributed to roughly $200,000 each year [37]. These costs appear to be much higher than the 90th percentile of costs among surgical NEC infants that we observed in the Medicaid cohort. The higher costs in the Spencer, et al. study are partly due to the use of billable Medicaid charges for home healthcare services, whereas we used the actual Medicaid paid amounts which typically represent very low reimbursement rates for these services. Also, children with SBS represent those who have the most severe health status among surgical NEC survivors. Our surgical NEC sample had 13 out of 62 patients (21%) between 6–12 months and 7 out of 35 patients (20%) between 24–36 months receiving care for intestinal malabsorption and the average costs in this sub-group would have been much higher. Also, it could not be ascertained from the claims data how many patients had severe SBS as defined in the Spencer, et al. study (i.e. loss of ≥ 70% of small intestinal length, ≥ 2 months of parenteral nutrition dependence, etc.).

Regardless of the data source, the healthcare costs reported in this paper are to be treated as highly conservative, since very expensive treatments such as small bowel transplantation were not accounted for in the analyses (because these procedures were not covered under Texas Medicaid during the study period). Transplant procedures may be required for long term survival in a small proportion of surgical NEC survivors with failure of intestinal function [38]. Also, a significant proportion of surgical NEC infants in the Medicaid sample were found to have NDD and the odds ratios were comparable to those reported in other studies [15-17]. The extent to which NDD influences direct healthcare costs is not very clear, although NDD can have a significant impact on diagnostic screening tests, physical and occupational therapy, and special education costs. More research using multiple data sources is needed to specifically understand the economic impact of neurodevelopmental delay among survivors of surgical NEC.

While the healthcare costs discussed above are highly relevant from a payer perspective, it should be remembered that the long term costs from a societal perspective would also account for the value of lives lost due to mortality attributed to NEC during the first 6 months of life. The value of a statistical life (VSL) is estimated at $7.4 million according to the 2002 Environmental Protection Agency (EPA) estimates [39]. Medicaid covers 40% of live births in the U.S. If the NEC mortality rate observed in the current study were to be applied to the overall Medicaid population in the U.S., approximately 700–900 Medicaid infants would be expected to die because of NEC annually and the total economic value of lives lost to NEC would be 5.2 – 6.6 billion US$.

This paper investigates the real world utilization of a longitudinal cohort of NEC survivors. Unfortunately, attrition in the sample resulted in smaller cohorts over time. However, the extent of attrition in the NEC and control groups was comparable and the balance in the baseline characteristics between the NEC and control cohorts was maintained over time. Additionally, a set of exploratory analyses (not shown in this paper) that was conducted to evaluate the probability of attrition over time showed that demographics and health outcomes in the previous time periods (e.g., healthcare costs, disability status and hospitalizations) together explained less than 10% of total variation in attrition in the Medicaid cohort. This suggests that attrition in the Medicaid cohort is predominantly caused by extrinsic factors that do not impact health outcomes (e.g., loss of Medicaid eligibility due to income changes, availability of employer insurance, migration to a different state, etc.)

A significant limitation of this study could be that our findings may not be generalizable to the universe of NEC survivors. This is because the study’s findings were derived from a sample of children that belonged to low-income families with a higher proportion of Hispanic children and, possibly, with a higher baseline risk for poor health status than NEC survivors in the commercially insured population. Additional research using healthcare utilization data obtained from a representative sample of commercially insured NEC survivors is needed in order to improve the generalizability of our findings. Nevertheless, given the fact that Medicaid is one of the largest payers of healthcare for children in the US, the estimates from this population are very useful in understanding the overall economic burden of surgical NEC from a US public payer perspective. Besides generalizability, the study also suffers from some of the classical limitations of using claims data that are not collected for research purposes. Most importantly, these data do not contain specific information that may be of potential research interest, such as gestational age of infants at birth, birth order, maternal characteristics such as education, income and breast feeding practices. Considering that data on the NEC population may be significantly hard to find in practice or simply too expensive to collect prospectively, we consider that the benefit of finding easy to collect longitudinal economic data outweighs the significant challenges of using retrospective claims data. Further research is needed on long term costs that are not captured by medical claims, such as special education costs and caregiver productivity costs.

## Conclusions

The healthcare costs of children who survived surgical necrotizing enterocolitis during birth hospitalization are substantial over the early childhood development period. Understanding the economic burden of NEC in the long term would aid healthcare providers, policy makers and payers to make informed decisions in providing care for infants at high risk for NEC. Further research on this topic is needed.

## Competing interests

This research was funded by an unrestricted educational grant from Prolacta Bioscience, CA, USA. Prof. Joel W. Hay has consulted in the past with Prolacta for work unrelated to the contents of this manuscript. Prof. Jae H. Kim has had a past affiliation with Prolacta Bioscience through support received for an industry-initiated clinical research study and through unrestricted research funds to the Department of Pediatrics, University of California, San Diego. Dr. Martin Lee and Dr. David Rechtman are current employees of Prolacta Bioscience.

## Authors’ contributions

Dr. Hay is the chief investigator for this research study and was responsible for providing overall supervision and guidance in the design, analyses and preparation of this manuscript. Vaidy Ganapathy is a doctoral candidate in Pharmaceutical economics & policy at USC who conducted the analyses and prepared a first draft of this manuscript. Dr. Kim provided expert support with the clinical aspects of the research. Dr. Lee and Dr. Rechtman provided significant comments on the first draft of the manuscript and Dr. Lee provided advice on the statistical analyses. All authors read and approved the final manuscript.

## Pre-publication history

The pre-publication history for this paper can be accessed here:

http://www.biomedcentral.com/1471-2431/13/127/prepub

## References

[B1] Neu J Walker WA Necrotizing enterocolitis New Eng J Med 2011 364 255 264 10.1056/NEJMra1005408 21247316 PMC3628622

[B2] Ng S Necrotizing enterocolitis in the full-term neonate J Paediatr Child Health 2001 37 1 4 11168859 10.1046/j.1440-1754.2001.00584.x

[B3] Pierro A Hall N Surgical treatments of infants with necrotizing enterocolitis Seminars in Neonatology : SN 2003 8 223 232 10.1016/S1084-2756(03)00025-3 15001141

[B4] Petrosyan M Guner YS Williams M Grishin A Ford HR Current concepts regarding the pathogenesis of necrotizing enterocolitis Pediatr Surg Int 2009 25 309 318 10.1007/s00383-009-2344-8 19301015

[B5] Horbar JD Carptenter JH 2007 Very low birth weight database summary 2008 Burlington, VT: Vermont Oxford Network

[B6] Fanaroff AA Hack M Walsh MC The NICHD neonatal research network: changes in practice and outcomes during the first 15 years Semin Perinatol 2003 27 281 287 10.1016/S0146-0005(03)00055-7 14510318

[B7] Grave GD Nelson SA Walker WA Moss RL Dvorak B Hamilton FA Higgins R Raju TNK New therapies and preventive approaches for necrotizing enterocolitis: report of a research planning workshop Pediatr Res 2007 62 510 514 10.1203/PDR.0b013e318142580a 17667844

[B8] Bell MJ Ternberg JL Feigin RD Keating JP Marshall R Barton L Brotherton T Neonatal necrotizing enterocolitis. Therapeutic decisions based upon clinical staging Ann Surg 1978 187 1 7 10.1097/00000658-197801000-00001 413500 PMC1396409

[B9] Cikrit D West KW Schreiner R Grosfeld JL Long-term follow-up after surgical management of necrotizing enterocolitis: sixty-three cases J Pediatr Surg 1986 21 533 535 10.1016/S0022-3468(86)80227-5 3723306

[B10] Bisquera JA Cooper TR Berseth CL Impact of necrotizing enterocolitis on length of stay and hospital charges in very low birth weight infants Pediatrics 2002 109 423 428 10.1542/peds.109.3.423 11875136

[B11] Ladd AP Rescorla FJ West KW Scherer L Engum SA Grosfeld JL Long-term follow-up after bowel resection for necrotizing enterocolitis: factors affecting outcome J Pediatr Surg 1998 33 967 972 10.1016/S0022-3468(98)90516-4 9694079

[B12] Blakely ML Gupta H Lally KP Surgical management of necrotizing enterocolitis and isolated intestinal perforation in premature neonates Semin Perinatol 2008 32 122 126 10.1053/j.semperi.2008.01.008 18346536

[B13] Simon NP Follow-up for infants with necrotizing enterocolitis Clinics In Perinatol 1994 21 411 424 8070234

[B14] Tejani A Dobias B Nangia BS Mahadevan R Growth, health, and development after neonatal gut surgery: a long-term follow-up Pediatrics 1978 61 685 693 96416

[B15] Hintz SR Kendrick DE Vohr BR Poole WK Higgins RD Changes in neurodevelopmental outcomes at 18 to 22 months’ corrected age among infants of less than 25 weeks’ gestational age born in 1993–1999 Pediatrics 2005 115 1645 1651 10.1542/peds.2004-2215 15930228

[B16] Schulzke SM Deshpande GC Patole SK Neurodevelopmental outcomes of very low-birth-weight infants with necrotizing enterocolitis: a systematic review of observational studies Arch Pediatr Adolescent Med 2007 161 583 590 10.1001/archpedi.161.6.583 17548764

[B17] Rees CM Pierro A Eaton S Neurodevelopmental outcomes of neonates with medically and surgically treated necrotizing enterocolitis Arch Dis Child Fetal Neonatal Ed 2007 92 F193 F198 10.1136/adc.2006.099929 16984980 PMC2675329

[B18] Medicaid coverage of births in Texas (March Of Dimes) Downloaded from: http://www.marchofdimes.com/peristats on 12/6./2012

[B19] Janek KL Ghahremani K Texas Medicaid Managed Care Texas Medicaid and CHIP in Perspective, Chapter 7 2013 Austin, TX: Texas Health and Human Services Commission

[B20] Rosenbaum PR Rubin DB Constructing a control group using multivariate matched sampling methods that incorporate the propensity score Am Stat 1985 39 33 38

[B21] Imbens GW Rubin DB Design in observational studies: matching to ensure balance in covariate distributions Causal Inference Part II, Chapter 15 2009 Cambridge University Press

[B22] Coca-Perraillon M Local and global optimal propensity score matching Statistics and Data Analysis. SAS Global Forum 2007 1 9 Downloaded from: http://www2.sas.com/proceedings/forum2007/185-2007.pdf

[B23] Mantel N Haenszel W Statistical aspects of the analysis of data from retrospective studies of disease J National Canc Inst 1959 22 719 748 13655060

[B24] Manning WG Mullahy J Estimating log models: to transform or not to transform? J Health Econ 2001 20 461 494 10.1016/S0167-6296(01)00086-8 11469231

[B25] Williams R Using the margins command to estimate and interpret adjusted predictions and marginal effects Stata J 2012 12 308 331

[B26] Wilmore DW Factors correlating with a successful outcome following extensive intestinal resection in newborn infants J Pediatr 1972 80 88 95 10.1016/S0022-3476(72)80459-1 4552656

[B27] Georgeson KE Breaux CW Outcome and intestinal adaptation in neonatal short-bowel syndrome J Pediatr Surg 1992 27 344 350 10.1016/0022-3468(92)90859-6 1501009

[B28] Goulet OJ Revillon Y Jan D De-Potter S Maurage C Lortat-Jacob S Martelli H Nihoul-Fekete C Ricour C Neonatal short bowel syndrome J Pediatr 1991 119 18 23 10.1016/S0022-3476(05)81032-7 1906099

[B29] Ricketts RR Jerles ML Neonatal necrotizing enterocolitis: experience with 100 consecutive surgical patients World J Surg 1990 14 600 605 10.1007/BF01658800 2238659

[B30] De-Souza JC Da-Motta UI Ketzer CR Prognostic factors of mortality in newborns with necrotizing enterocolitis submitted to exploratory laparotomy J Pediatr Surg 2001 36 482 486 10.1053/jpsu.2001.21603 11227002

[B31] Dzakovic A Notrica DM Smith EO Wesson DE Jaksic T Primary peritoneal drainage for increasing ventilatory requirements in critically ill neonates with necrotizing enterocolitis J Pediatr Surg 2001 36 730 732 10.1053/jpsu.2001.22947 11329576

[B32] Adams-Chapman I Stoll BJ Neonatal infection and long-term neurodevelopmental outcome in the preterm infant Curr Opin Infect Dis 2006 19 290 297 10.1097/01.qco.0000224825.57976.87 16645492

[B33] Ganapathy V Hay JW Kim JH Costs of necrotizing enterocolitis and cost-effectiveness of exclusively human milk-based products in feeding extremely premature infants Breastfeed Med 2012 7 1 29 37 10.1089/bfm.2011.0002 21718117

[B34] Russell RB Green NS Steiner CA Meikle S Howse JL Poschman K Dias T Potetz L Davidoff MJ Damus K Petrini JR Cost of hospitalization for preterm and low birth weight infants in the United States Pediatrics 2007 120 e1 e9 10.1542/peds.2006-2386 17606536

[B35] Underwood MA Danielsen B Gilbert WM Cost, causes and rates of rehospitalization of preterm infants J Perinatol 2007 27 614 619 10.1038/sj.jp.7211801 17717521

[B36] Hamrick SEG Hansmann G Patent ductus arteriosus in the preterm infant Pediatrics 2010 125 19 26 10.1542/peds.2009-1878D 20421261

[B37] Spencer AU Kovacevich D McKinney-Barnett M Hair D Canham J Maksym C Teitelbaum DH Pediatric short-bowel syndrome: the cost of comprehensive care Am J Clin Nutr 2008 88 1552 1559 10.3945/ajcn.2008.26007 19064515

[B38] Vennarecci G Kato T Misiakos EP Neto AB Verzaro R Pinna A Nery J Khan F Thompson JF Tzakis AG Intestinal transplantation for short gut syndrome attributable to necrotizing enterocolitis Pediatrics 2000 105 E25 10.1542/peds.105.2.e25 10654985

[B39] Johansson P On the definition and estimation of the value of a statistical life. Departmental Working Papers 2006-23, Department of Economics, Management and Quantitative Methods at Universita degli Studi di Milano Downloaded from: http://wp.demm.unimi.it/tl_files/wp/2006/DEMM-2006_023wp.pdf

